# Hierarchy Low CD4+/CD8+ T-Cell Counts and IFN-γ Responses in HIV-1+ Individuals Correlate with Active TB and/or *M*.*tb* Co-Infection

**DOI:** 10.1371/journal.pone.0150941

**Published:** 2016-03-09

**Authors:** Lingyun Shao, Xinyun Zhang, Yan Gao, Yunya Xu, Shu Zhang, Shenglei Yu, Xinhua Weng, Hongbo Shen, Zheng W. Chen, Weimin Jiang, Wenhong Zhang

**Affiliations:** 1 Department of Infectious Diseases, Huashan Hospital, Fudan University, Shanghai, 200040, China; 2 Department of Infectious Diseases, Honghe No.1 People’s Hospital, Mengzi, Mengzi County, 661100, China; 3 Chinese Academy of Science and Institute Pasteur of Shanghai, Shanghai, 200040, China; 4 Department of Microbiology & Immunology, Center for Primate Biomedical Research, University of Illinois College of Medicine, Chicago, Illinois, 60612, United States of America; National AIDS Research Institute, INDIA

## Abstract

**Objective:**

Detailed studies of correlation between HIV-*M*.*tb* co-infection and hierarchy declines of CD8+/CD4+ T-cell counts and IFN-γ responses have not been done. We conducted case-control studies to address this issue.

**Methods:**

164 HIV-1-infected individuals comprised of HIV-1^+^ATB, HIV-1^+^LTB and HIV-1^+^TB^-^ groups were evaluated. Immune phenotyping and complete blood count (CBC) were employed to measure CD4+ and CD8+ T-cell counts; T.SPOT.TB and intracellular cytokine staining (ICS) were utilized to detect ESAT6, CFP10 or PPD-specific IFN-γ responses.

**Results:**

There were significant differences in median CD4+ T-cell counts between HIV-1^+^ATB (164/μL), HIV-1^+^LTB (447/μL) and HIV-1^+^TB^-^ (329/μL) groups. Hierarchy low CD4+ T-cell counts (<200/μL, 200-500/μL, >500/μL) were correlated significantly with active TB but not *M*.*tb* co-infection. Interestingly, hierarchy low CD8+ T-cell counts were not only associated significantly with active TB but also with *M*.*tb* co-infection (*P*<0.001). Immunologically, HIV-1^+^ATB group showed significantly lower numbers of ESAT-6-/CFP-10-specific IFN-γ+ T cells than HIV-1^+^LTB group. Consistently, PPD-specific IFN-γ+CD4+/CD8+ T effector cells in HIV-1^+^ATB group were significantly lower than those in HIV-1^+^LTB group (*P*<0.001).

**Conclusions:**

Hierarchy low CD8+ T-cell counts and effector function in HIV-1-infected individuals are correlated with both *M*.*tb* co-infection and active TB. Hierarchy low CD4+ T-cell counts and Th1 effector function in HIV-1+ individuals are associated with increased frequencies of active TB, but not *M*.*tb* co-infection.

## Introduction

Globally, tuberculosis (TB) may affect up to 30% of an estimated 34 million people living with HIV-1 infection and indeed is the leading cause of mortality in HIV-1-infected persons [1, 2]. While HIV-1 infection is the leading risk factor for developing active TB, ~5–15% of HIV-1 cases annually develop TB by reactivating latent *Mycobacterium tuberculosis* (*M*.*tb*) infection, and ~90% of HIV-1-infected persons can develop TB during their lifetime [3].

HIV infection clearly increases susceptibility to TB [4, 5], but relative importance of HIV infection, CD4+ T cell depletion or both have not been elucidated. Recent mechanistic studies in nonhuman primates have demonstrated that CD4+ T cells are clearly needed to control TB infection and sustain multi-effector function of CD8+ T cells and other immune cells [6]. Although evidence that CD8+ T cells protect against TB is lacking in humans and controversial in mice [4], primate CD8+ T cells have been shown to be critical for immunity against TB [7]. Thus, immunological studies of correlation between *M*.*tb* co-infection/TB and CD4+ and CD8+ T cells in HIV-1-infected humans will ultimately help define anti-TB immunity and mechanisms of these T-cell populations.

CD4+ T-cell count ≤200/μL is defined as acquired immunodeficiency syndrome (AIDS) and highly susceptible to TB and opportunistic infections [2, 8, 9]. However, little is known about what extent to which CD8+ T-cell counts and effector functions decline in HIV-1-infected humans can impact *M*.*tb* co-infection or active TB. Although active anti-viral therapy (ART) reduces opportunistic infections in HIV-infected patients [10, 11], the increased risk of TB conferred by HIV infection does not appear to be significantly diminished by ART. Therefore, further studies are needed to determine what levels of CD8+/CD4+ T cells and their effector functions during ART and prolonged residual HIV infection still predispose HIV-1-infected individuals to developing *M*.*tb* co-infection or active TB. To address these questions, we recruited 164 HIV-1-infected individuals with different statuses of *M*.*tb* co-infection and evaluated whether hierarchy declines of CD4+ and CD8+ T-cell counts and effector functions correlated with *M*.*tb* co-infection and active TB.

## Methods

### Study participants

One hundred and sixty-four HIV-1-infected individuals from Yunnan Province and Shanghai were recruited in this study from 2010 to 2012. All HIV-1-infected individuals were confirmed by clinical data, routine serum detection (competitive ELISA and Western blotting confirmation), CD4+ and CD8+ T-cell counts. CD4+ and CD8+ T cells were identified and determined using the CD3/CD4/CD8 Tritest kit (BD Biosciences, CA) by following the manufacturer’s manual. Information on the following variables was collected by completing a detailed questionnaire: age, gender, BCG vaccination, TB history of prior active TB, chest radiography, sputum smear microscopy, sputum culture and other medical examination. All individuals had a history of newborn Bacille Calmette-Guerin (BCG) vaccination.

Subjects were divided into 3 groups according to the status of *M*.*tb* infection. (1). HIV-1^+^ATB group (HIV-1 co-infected with active TB; n = 30): active TB was diagnosed with the clinical evidence of TB including clinical TB symptoms, positive status of smear test for acid-fast bacilli from sputum and/or *M*.*tb* culture, and abnormal chest radiograph. (2). HIV-1^+^LTB group (HIV-1 co-infected with latent TB; n = 59): latent TB was diagnosed based on the findings that their T-SPOT.TB tests were positive, but without clinical manifestations of active pulmonary and extrathoracic TB, negative status for sputum smear and/or bacilli culture, and normal chest radiograph. (3). HIV-1^+^TB^-^ group: (HIV-1-infected only, without *M*.*tb* infection, n = 75): HIV-1+ individuals showed negative T-SPOT.TB test, with no evidence of TB. About <1/3 of subjects in each of groups received antiretroviral therapy (ART) according to 2012 DHHS Antiretroviral Therapy Guidelines (aidsinfo.nih.gov). Data of CD4+/CD8+ T-cell counts and antigen-specific IFN-γ responses between groups were not significantly different with or without ART (data not shown).

### Ethics statement

The study was approved with written consent by the Institutional Review Board of Fudan University, and written informed consent was obtained from all the participants.

### T-SPOT.TB assay

T-SPOT.TB assay was performed as previously described [12–14]. Peripheral blood mononuclear cells (PBMCs) were isolated from heparinized venous blood by Ficoll-Paque centrifugation. T-SPOT.TB kit (Oxford Immunotec Ltd., Oxford, UK), a novel commercial ELISPOT assay to detect IFN-γ release induced by *M*.*tb* ESAT-6 and CFP-10, was employed to identify *M*.*tb* infection including latent and active *M*.*tb* infection. The test result of T-SPOT.TB assay was considered positive if either or both of Panel A (containing peptide antigens derived from ESAT-6) or Panel B (containing peptide antigens derived from CFP-10) had six or more spots than the negative control, and this number was at least two times greater than the number of spots in the negative controls according to manufacturer's instructions. The spots were read using the ELISPOT plate reader (AID-Gmb-H, Germany).

### Immunofluorescent staining and flow cytometric analysis

For cell-surface staining, 100μL EDTA blood was treated with red blood cell (RBC) lysing buffer and washed twice with 5% fetal bovine serum (FBS)-phosphate-buffered saline (PBS) before staining. PBMCs were stained with up to four Abs (conjugated to FITC, PE, allophycocyanin, pacific blue, and PE-Cy5) for at least 10 min at room temperature. After staining, cells were fixed with 2% formaldehyde-PBS prior to analysis on a BD FACS Aria flow cytometer (BD Bioscience, San Diego, California, USA). Lymphocytes were gated based on forward-scatters and side-scatters; at least 20 000-gated events were analyzed using FCS Express V3 Software. Absolute cell numbers were calculated based on flow cytometry and CBC data. The following mouse anti-human mAbs were used: CD3 (SP34, SP34-2), CD8 (RPA-T8), IFN-γ (4S.B3), (BD Pharmingen, San Diego, California, USA); CD4 (OKT4) (eBioscience, SanDiego, Califonia, USA); CD8 (DK25) (Dako, Glostrup, Denmark).

### Intracellular cytokine staining (ICS)

ICS was performed as previously described [12]. Briefly, 10^6^ PBMCs plus costimulatory mAbs CD28 (1μg/mL) and CD49d (1 μg/mL) were incubated with PPD (25μg/mL) or media alone in 200 μL final volume for 1 h at 37°C, 5% CO_2_ followed by an additional 5 h incubation in the presence of brefeldin A (Golgi Plug; BD Bioscience). After stained with cell-surface CD3/CD4/CD8 for at least 15 min at room temperature, cells were permeabilized for 45 min (Cytofix/cytoperm; BD Bioscience) at 4°C and stained another 45 min for IFN-γ at room temperature before resuspending in 2% formaldehyde-PBS.

### Statistical analysis

The nonparametric quantitative variables across groups were compared by use of the Mann-Whitney test and qualitative variable by chi-square test. The relation between clinical and demographic characteristics was assessed by ANOVA. Receiver operating characteristic curve (ROC) analysis was conducted to determine the sensitivity and specificity with varying cut-off values for CD4+ and CD8+ T cell counts. The ROC curves of the CD4+ and CD8+ counts were compared by assessing equivalence of areas under the curve (AUCs). Significance was inferred for *P*<0.05 with two-sided, and all analyses were done with SPSS (version 19) software.

## Results

### The demographic and clinical characteristics of study subjects

A total of 164 HIV-1-infected individuals were recruited during the study period. Of the 164 participants, the T-SPOT.TB assay was positive in 79 (48.2%) individuals overall. Among 30 HIV-1-infected patients with active TB (ATB), 20 (66.7%) were T-SPOT.TB positive. The remaining 59 T-SPOT.TB positive individuals had no evidence of active TB, and were defined as LTB. The demographic profiles of all the subjects were shown in Table 1.

**Table 1:** Demographic and clinical characteristics of study subjects.

	Total	HIV-1^+^ATB group	HIV-1^+^LTB group	HIV-1^+^TB^-^ group	*P* value
**Patient number**	164	30	59	75	NA
**Male/female (% of male)**	110/164 (67%)	20/30 (66.7%)	37/59 (62.7%)	53/75 (70.6%)	0.811
**Median Age (year, Range)**	36 (14–73)	36 (26–64)	36 (24–73)	33 (14–56)	0.972
**Median CD4+ T cell counts (/μL, Range)**	335 (1–1359)	164 (3–586)	447 (41–1359)	329 (1–851)	<0.001
**Median CD8+ T cell counts (/μL, Range)**	527 (4–1782)	244 (4–977)	482 (108–1415)	659 (169–1782)	<0.001
**Previous TB history, n (%)**	9 (5.5%)	2 (6%)	1 (1.7%)	6 (8%)	0.270
**Patients on HARRT, n (%)**	43 (26.2%)	19 (63%)	13 (22.0%)	12 (16%)	<0.001
**HIV-1 viral load (copies/mL, Range)**	50 (50, 180000)	50 (50, 7000)	50 (50, 33000)	200 (3700, 180000)	0.222

NA: not applicable

### Hierarchy declines of CD4+ T-cell counts in HIV-1+ individuals are associated with increased frequencies of active TB, but not *M*.*tb* co-infection

**Fig 1:**
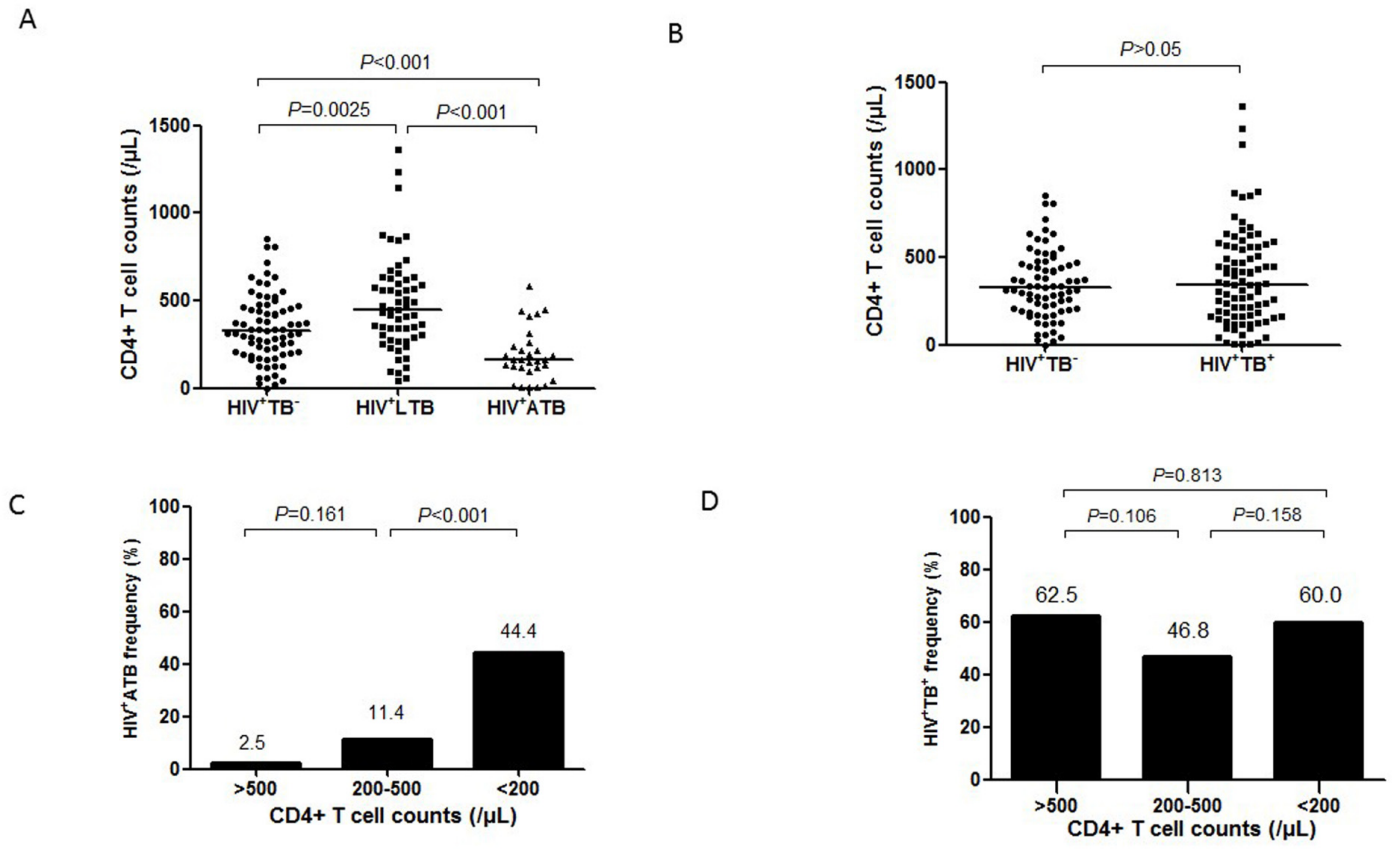
Hierarchy low CD4+ T-cell counts associated with active TB but not *M.tb* coinfection. **A**. CD4+ T cell counts in groups of HIV-1^+^TB^-^, HIV-1^+^LTB and HIV-1^+^ ATB. **B**. CD4+ T cell counts in HIV-1-infected individuals with or without *M*.*tb* co-infection (LTB+ATB). The horizontal lines represent the median CD4+ T cell counts in each group. **C and D**. Changes of the incidence of active TB (C.) and *M*.*tb* co-infection (D.) in HIV-1^+^ individuals with different CD4+ T cell counts. Data were analyzed using chi-square test and *P* values are indicated.

Although CD4+ T-cell counts of 200/μL are defined as HIV-induced AIDS [15], additional studies are needed to determine what declining levels of CD4+ T cells predisposes HIV-1-infected individuals to developing *M*.*tb* co-infection or active TB. In this study, we found that the median CD4+ T cell counts in HIV-1^+^ATB group (164/μL) were significantly lower than the HIV-1^+^TB^-^ (329/μL) and HIV-1^+^LTB (447/uL) (both *P*<0.001) (Fig 1A). Nevertheless, when we combined HIV-1^+^LTB and HIV-1^+^ ATB groups as *M*.*tb* co-infection group, we found no significant difference in CD4+ T-cell counts between HIV-1+ individuals with and without *M*.*tb* co-infection (*P*>0.05; Fig 1B). To rule out the influence of ART on the relationship between CD4+ T cell counts and *M*.*tb* co-infection status, we divided the subjects into ART and without ART subgroups according to the HIV treatment. We found that the median CD4+T cell counts showed similar tendency with the total study population with different *M*.*tb* co-infection status.

To examine if hierarchy declines of CD4+ T cells were associated with active TB, we divided all studied subjects into three CD4-quantitative groups based on levels of CD4+ T-cell counts: <200/μL (n = 45), 200-500/μL (n = 79) and >500/μL (n = 40). Interestingly, median frequencies of active TB were 44.4%, 11.4% and 2.5%, respectively, in the three groups whose CD4+ T cell counts were <200/μL, 200-500/μL and >500/μL, with the highest frequency in subjects with CD4+ T cell counts <200/μL (*P*<0.001; Fig 1C). Again, to our surprise, hierarchy declines of CD4+ T-cell counts did not coincide with *M*.*tb* co-infection (both latent and active), as median frequencies of *M*.*tb* infection were similarly at 60.0%, 46.8% and 62.5%, respectively, in these three groups (*P*>0.05; Fig 1D). Thus, these results suggest that hierarchy low CD4+ T-cell counts in HIV-1-infected humans are associated with active TB, but not *M*.*tb* infection.

### IFN-γ responses of *M*.*tb*-specific CD4+ T cells are much lower in HIV+ATB group than those in HIV+LTB group

**Fig 2:**
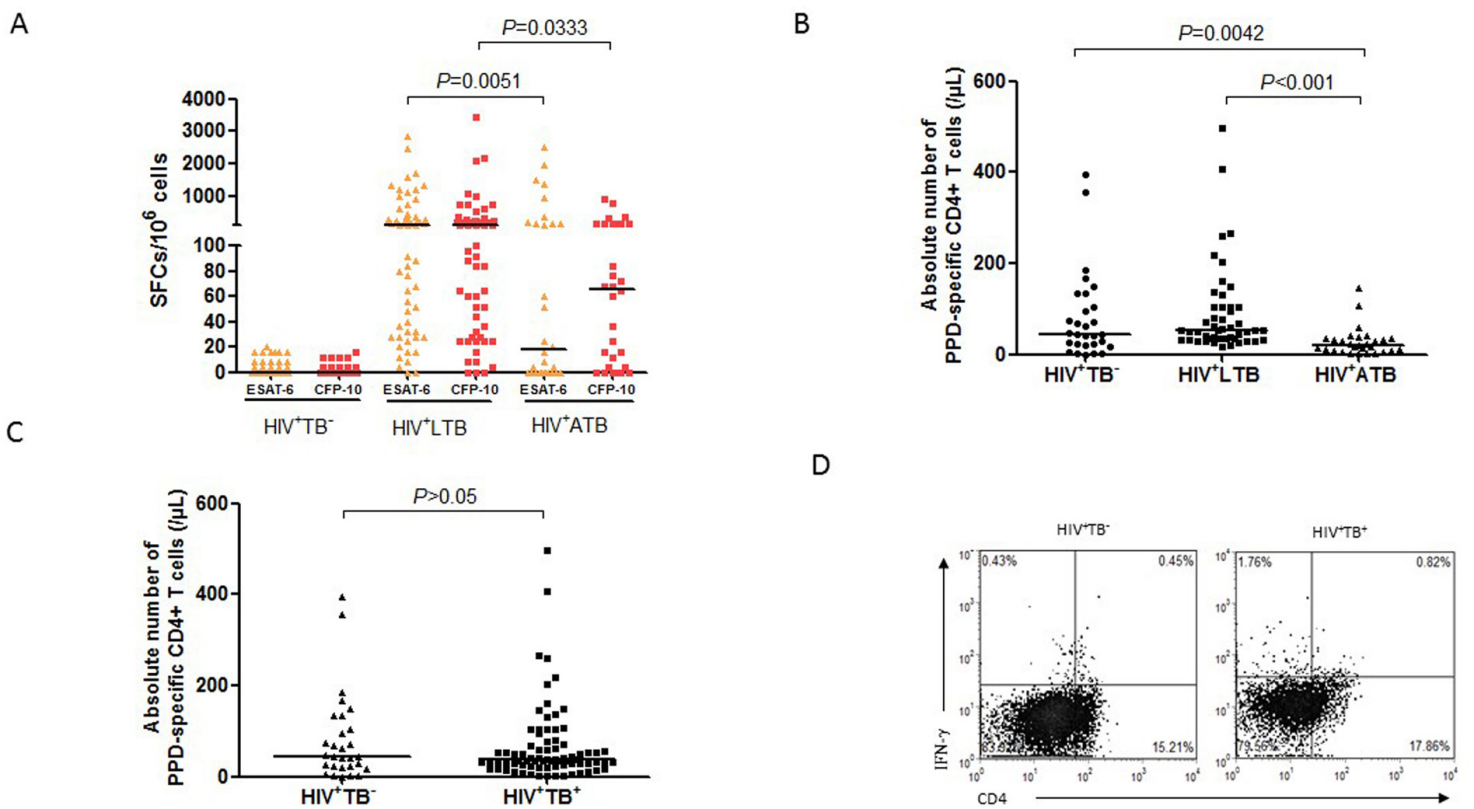
The absolute numbers of TB-specific IFN-γ+ cells and CD4+ T effector cells in HIV-1^+^ individuals with different TB infection status. **A**. Spot-Forming Cells (SFCs) of ESAT6-/CFP10-specific IFN-γ+ T cells in PBMCs in groups of HIV-1^+^ATB, HIV-1^+^ LTB and HIV-1^+^TB^-^. **B**. Numbers of PPD-specific IFN-γ+CD4+ T cells in groups of HIV-1^+^ATB, HIV-1^+^ LTB and HIV-1^+^TB^-^. **C**. Numbers of PPD-specific IFN-γ+CD4+ T cells in HIV-1^+^ individuals with or without *M*.*tb* co-infection (LTB+ATB). The horizontal lines represent the medians of cell numbers for each group. **D**. Representative CD3-gated histograms of PPD-specific IFN-γ+CD4+ T cells from cases of HIV-1^+^TB^+^ group and HIV-1^+^TB^-^ group.

We then examined if numbers of *M*.*tb*-specific CD4+ T effector cells similarly correlated with active TB in HIV-1-infected individuals. We first measured *M*.*tb* ESAT6- and CFP10-specific IFN-γ+ T effector cells in the three groups of HIV-1-infected individuals using ELISPOT assay (T-SPOT.TB) as reported studies showed that CD4+ T effector cells appeared to be more dominant than CD8+ T cells within these peptide-specific T-cell population during *M*.*tb* infection. Interestingly, most HIV+LTB subjects exhibited detectable immune response of ESAT-6/CFP-10-specific IFN-γ+ T cells in PBMCs, with median numbers of >150 spots/10^6^ cells after stimulation with ESAT-6 or CFP-10 (Fig 2A). In contrast, levels of these T effector cells were significantly lower in HIV+ATB group (Fig 2A, *P*<0.0333 for CFP-10, *P*<0.0051 for ESAT-6). Overall, ESAT-6-specific T-cell response in HIV+ATB group was low regardless of ART (data not shown). Consistently, when we utilized ICS to measure PPD-specific Th1 cells, we found that frequencies of PPD-specific IFN-γ+ CD4+ T cells in HIV-1+LTB group were significantly higher than HIV-1^+^ATB and HIV-1^+^TB^-^ groups (Fig 2B, *P*<0.001 and *P* = 0.0042, respectively). Of note, low-level PPD-specific CD4+ T cells in HIV^+^TB^-^ group might reflect some degree of immune response after BCG vaccination (Fig 2B). However, there was no significant difference in PPD-specific CD4+ T-cell responses between HIV-1^+^TB^-^ and combined HIV+ATB/HIV+LTB (both active/latent TB, *P*>0.05; Fig 2C and 2D). These results therefore suggest that immune responses of *M*.*tb*-specific CD4+ T cells are much lower in HIV+ATB group than those in HIV+LTB group.

### Hierarchy low CD8+ T-cell counts in HIV-1 infection are associated with both *M*.*tb* co-infection and active TB

**Fig 3:**
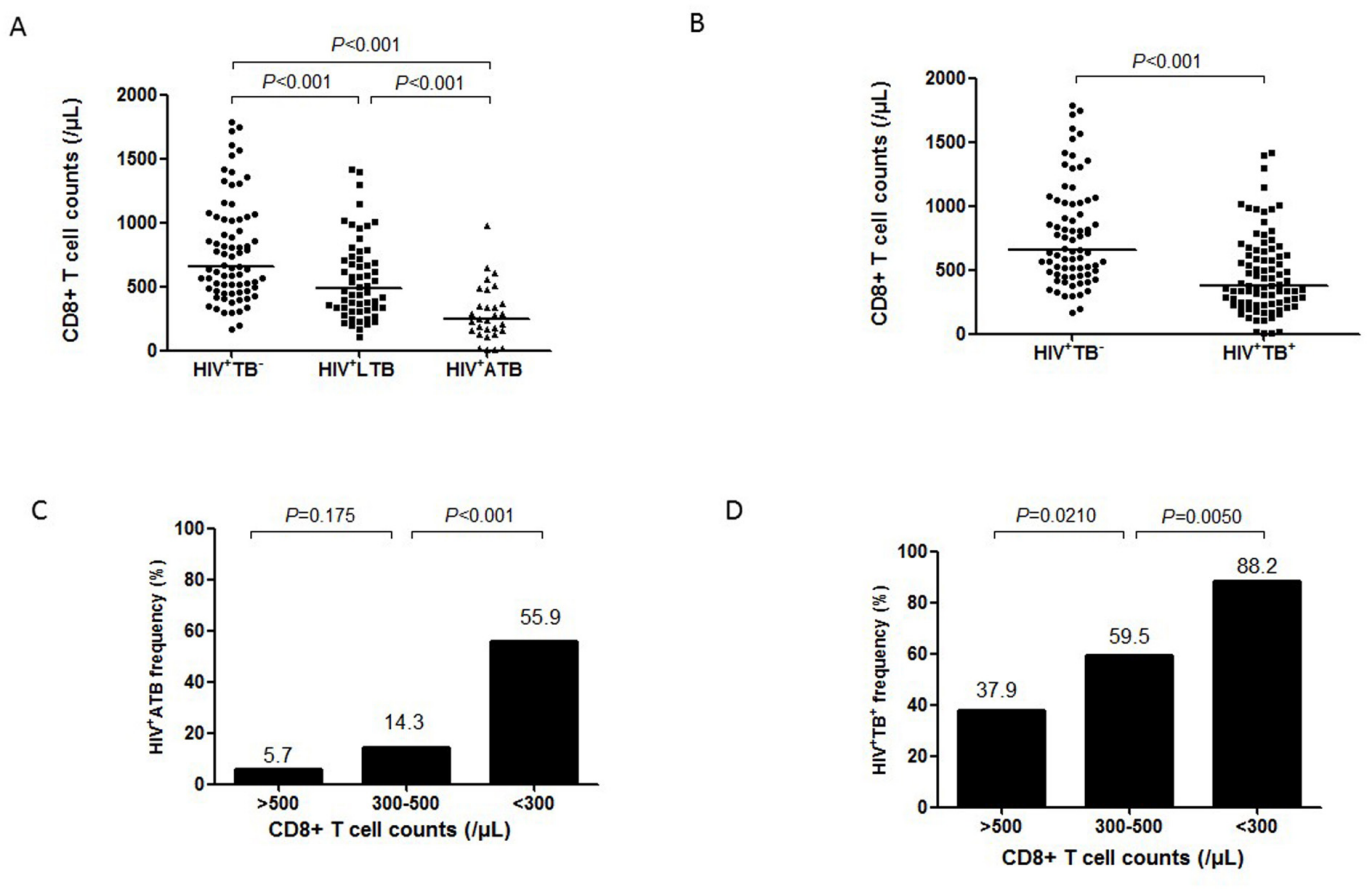
CD8 T-cell counts in HIV-1-infected individuals with different *M*.*tb* infection status. **A**. CD8+ T-cell counts in groups of HIV-1^+^TB^-^, HIV-1^+^LTB and HIV-1^+^ ATB. **B**. CD8+ T cell counts in HIV-1-infected individuals with or without *M*.*tb* co-infection. The horizontal lines represent the median of CD8+ T cell counts in each group. **C**. **and D**. Frequencies of active TB (C.) and *M*.*tb* co-infection (D.) in HIV-1^+^ individuals with different CD8+ T-cell counts. Data were analyzed using chi-square test and *P* values are indicated.

Although HIV-1 infection can stimulate increases in immune responses of CD8+ T cells, numbers and function of CD8+ T cells may decline with exhaustion during progression of HIV/AIDS [16]. Virtually, whether declined numbers of CD8+ T cells correlate with HIV-related TB has not been demonstrated. We addressed this scientific question as we found that many HIV-1-infected persons had significant lower CD8+ T-cell counts. This could not be explained by simple ART suppression of HIV replication, but might be related to progression of prolonged residual HIV-1 infection. We previously demonstrated that CD8+ T cells play a critical role in anti-TB immunity in immune competent individuals [7]. We therefore hypothesize that decreased numbers of CD8+ T cells during progression of HIV/AIDS may increase risks for *M*.*tb* co-infection and active TB. We found that CD8+ T-cell counts were not only associated with active TB disease but also with *M*.*tb* co-infection including both latent and active status since median numbers of CD8+ T cells were 244/μL (CD4, 168), 482/μL (CD4, 447) and 659/μL (CD4, 329), respectively, in HIV-1^+^ATB, HIV-1^+^LTB and HIV-1^+^TB^-^ groups (all *P*<0.001; Fig 3A). Consistently, CD8+ T-cell counts in the combined HIV-1^+^ATB/HIV-1^+^LTB group (active/latent *M*.*tb* co-infection) were significantly lower than HIV-1^+^TB^-^ group, with the median of 379/μL and 659/μL, respectively (*P*<0.001; Fig 3B).

Next, we sought to examine if hierarchy low CD8+ T-cell counts correlated with increased occurrence of *M*.*tb* infection and active TB using receiver operating characteristics (ROC) curve (S1 Fig). We divided all HIV-1-infected subjects into three CD8-quantitative groups based on the CD8+ T-cell counts: <300/μL (n = 34), 300-500/μL (n = 42) and >500/μL (n = 87). Surprisingly, the frequencies of ATB and LTB were associated with the hierarchy low CD8+ T-cell counts (Fig 3C). Median frequencies of TB were 55.9%, 14.3% and 5.7%, respectively, in the three CD8-quantitative groups, with the highest one in subjects with CD8+ T cell counts <300/μL (*P*<0.001; Fig 3C). When active/latent *M*.*tb* co-infection was calculated, median frequencies of *M*.*tb* co-infection were 88.2%, 59.5% and 37.9%, respectively, in the three CD8-quantitative groups (both *P*<0.05; Fig 3D). Thus, CD8+ T cells are associated with both *M*.*tb* co-infection and active TB, and hierarchy low CD8+ T-cell counts in HIV-1 infection correlate with increased frequencies of *M*.*tb* co-infection and active TB.

### Low immune responses of Ag-specific CD8+ T cells were associated with *M*.*tb* co-infection in HIV-1-infected subjects

**Fig 4:**
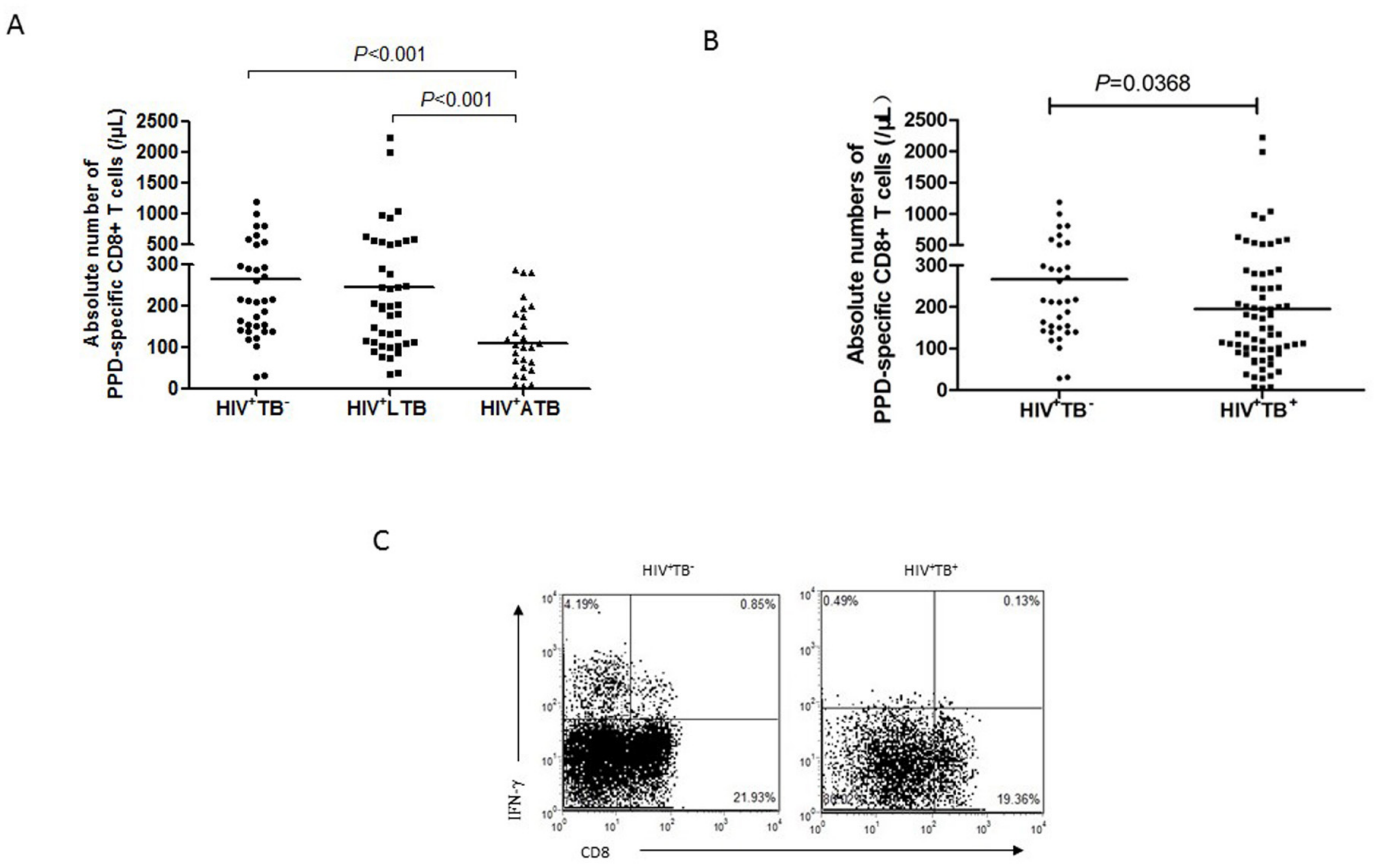
The absolute numbers of PPD-specific IFN-γ+ CD8+ T cells in HIV-1^+^ individuals with or without *M*.*tb* co-infections. **A**. Absolute numbers of PPD-specific IFN-γ+CD8+ T cells in groups of HIV-1^+^ATB, HIV-1^+^ LTB and HIV-1^+^TB^-^. **B**. Absolute numbers of PPD-specific IFN-γ+CD8+ T cells in HIV-1^+^ individuals with or without *M*.*tb* co-infection. The horizontal lines represent the medians of cell numbers for each group. **C**. Representative CD3-gated histograms of PPD-specific IFN-γ+CD8+ T cells from cases of HIV-1^+^TB^-^ group and HIV-1^+^TB^+^ group.

Our previous study in smaller cohorts demonstrated that higher frequencies of γδ and CD8+ T effector cells correlated with latent status of *M*.*tb* infection [12]. Here, we recruited more HIV-1-infected persons to address a new question as to if low Ag-driven CD8+ T effector cells in HIV-1-infected humans were associated with both active TB and *M*.*tb* co-infection. Numbers of PPD-specific IFN-γ+ CD8+ T cells in HIV-1+ATB were significantly lower than HIV+LTB and HIV+TB- groups (Fig 4A, both *P*<0.001). Notably, detectable PPD-specific CD8+ T cells in HIV+TB- group might represent memory response after BCG vaccination as well as innate-like CD8+ T effector response. We were therefore interested in comparative analysis of this effector response in HIV-1+ individuals with and without *M*.*tb* co-infection. Our data showed that IFN-γ+ CD8+ T effector cells in the combined HIV-1^+^ATB/HIV-1^+^LTB group (active/latent *M*.*tb* co-infection) were significantly lower than HIV+TB- group (*P* = 0.0368; Fig 4B and 4C). Thus, the current study confirms our previous finding that robust CD8+ T cell response in HIV+ persons is associate with LTB [12], the new data establish association between CD8+ T effector function and *M*.*tb* co-infection in HIV-1-infected individuals.

## Discussion

The current study represents a first detailed analysis of CD8+ T-cell count association with *M*.*tb* co-infection and active TB in HIV-1-infected humans. We extend previous work [12] to demonstrate that hierarchy low CD8+ T-cell counts and antigen-specific CD8+ T-cell response correlates not only with active TB but also with *M*.*tb* co-infection in HIV-1-infected patients. In addition, our study confirms the published findings that HIV infection increases in risks for TB, but also provides new association data suggesting that hierarchy low CD4+ T-cell counts and Th1 effector function correlate with active TB, but not co-infection, in HIV-1-infected persons. It is worth to mention that due to short-term ART in about <1/3 patients in each group, data of CD4+/CD8+ T-cell counts and antigen-specific IFN-γ responses were not significantly different between the groups with or without ART (data not shown).

Hierarchy low CD4+ T-cell counts and Th1 effector function have not been well assessed for association with *M*.*tb* co-infection and active TB in HIV-1-infected persons [4, 12, 17–20]. We find that as high as 44% of HIV-1-infected persons whose CD4+ T cell counts are <200/μL develop active TB after *M*.*tb* co-infection. Consistently, Ag-specific CD4+ T effector cells in HIV-1+ATB group are also lower than both HIV+LTB and HIV+TB- groups (*P*<0.001 and *P* = 0.0042, respectively). These results are consistent with the concept that CD4+ T-cell count <200/μL is the definition point for HIV/AIDS associated with an increased risk of TB and other opportunistic infections [12, 21]. Notably, we provide additional information that hierarchy low CD4+ T-cell counts are not associated with *M*.*tb* co-infection including latent and active TB status. Our finding suggests that CD4+ T-cell counts appear to be a determining factor for active TB rather than *M*.*tb* co-infection. The results consist with recent findings that CD4+ T cells are required to contain extrapulmonary TB and rapid TB progression after infection [5, 6, 22].

Interestingly, we find that hierarchy declines of CD8+ T-cell counts are associated with both *M*.*tb* co-infection and active TB in HIV-1-infected humans (*P*<0.001). Further analysis indicates that the hierarchy low CD8+ T cells in HIV-1+ individuals coincide with increased frequencies of *M*.*tb* co-infection including ATB and LTB. Strikingly, 88% of HIV-1-infected humans whose CD8+ T cell counts are ≤ 300/μL develop *M*.*tb* co-infection, and this frequency is significantly higher than HIV+LTB and HIV+TB- groups. These results suggest that a decline of CD8+ T-cell counts during progression of HIV-1 infection might be a risk factor for susceptibility to *M*.*tb* co-infection.

Of note, CD4+/CD8+ ratios in our cohorts of HIV-1 infected individuals remain inverse or low mostly due to low CD4+ T cell counts despite early ART (data not shown). The reason for decreases in absolute numbers of CD8+ T cells in these subjects may be two-fold. The decline can certainly occur as a result of a reduced immune stimulation of CD8+ T cells by lower HIV viral loads during ART. One would expect that such decline of absolute CD8+ T-cell count would not be dramatic if immune restoration by ART is apparent despite CD4+/CD8+ ratios are significantly reduced. On the other hand, significant decreases in numbers of CD8+ T cells might result primarily from weaken immune homeostasis due to prolonged and residual HIV infection. This notion is supported by our recent observation that CD4+ T-cell depletion leads to subsequent losses of CD8+ T-cell counts and immune responses [6].

The current study involving more HIV+ individuals demonstrates that detectable immune response of PPD-specific CD8+ T cells appears to be one of surrogate markers for *M*.*tb* co-infection including LTB and ATB in HIV-1-infected humans as HIV+TB- group exhibits no or few PPD-specific CD8+ T cells. Consistent with the previous observation that Ag-specific CD8+ T-cell responses in HIV+LTB subjects are more robust than those in HIV+ATB patients [12], the hierarchy decline of CD8+ T cell counts to ≤300/μL in HIV+ subject is associated with an increased rate of *M*.*tb* co-infection. Taken together, the results from two separate studies suggest that reduced CD8+ T-cell counts and IFN-γ production by CD8+ T cells might predispose HIV-1-infected humans to *M*.*tb* co-infection and active TB. To date, it has remained unknown whether CD8+ T cells contribute to anti-TB immunity against *M*.*tb* co-infection or active TB in HIV-1-infected persons. We recently found that primate CD8+ T cells and their effector function are critical for controlling *M*.*tb* infection and TB lesions [7, 23], but sustaining CD8+ T-cell responses relies on helper function of CD4+ T cells [6]. Consistently, the depletion of TNF-α+ CD8^+^ T cells by anti-TNF-α immunotherapy may contribute to reactivation TB in humans [24].

Thus, hierarchy low CD4+ T cell counts and Th1 cells in HIV-1-infected humans correlate with increased occurrence of active TB, but not *M*.*tb* co-infection. On the other hand, hierarchy low CD8+ T cell counts and their IFN-γ effector function in HIV-1-infected individuals are found coincident with *M*.*tb* co-infection and active TB. Our findings suggest that HIV-1-infected persons with CD4+ T-cell counts ≤200/μL and/or CD8+ T-cell counts ≤300/μL appear to represent high-risk populations with increased susceptibility to *M*.*tb* co-infection and/or active TB, and may require increased clinical monitoring and intervention.

## Supporting Information

S1 Fig Receiver operating characteristics (ROC) curve for differentiating M.tb infection and active TB with CD8+ T cell count cut-off of 300/μL. AUC: the area under the curve.
(TIF)

S1 Dataset The minimal data set of this study. (XLS)
